# Electronic Structure of Ternary Alloys of Group III and Rare Earth Nitrides

**DOI:** 10.3390/ma14154115

**Published:** 2021-07-23

**Authors:** Maciej J. Winiarski

**Affiliations:** Institute of Low Temperature and Structure Research, Polish Academy of Sciences, Okólna 2, 50-422 Wrocław, Poland; m.winiarski@int.pan.wroc.pl

**Keywords:** nitride semiconductors, band gap, calculations

## Abstract

Electronic structures of ternary alloys of group III (Al, Ga, In) and rare earth (Sc, Y, Lu) nitrides were investigated from first principles. The general gradient approximation (GGA) was employed in predictions of structural parameters, whereas electronic properties of the alloys were studied with the modified Becke–Johnson GGA approach. The evolution of structural parameters in the materials reveals a strong tendency to flattening of the wurtzite type atomic layers. The introduction of rare earth (*RE*) ions into Al- and In-based nitrides leads to narrowing and widening of a band gap, respectively. Al-based materials doped with Y and Lu may also exhibit a strong band gap bowing. The increase of a band gap was obtained for Ga${}_{1-x}$Sc${}_{x}$N alloys. Relatively small modifications of electronic structure related to a *RE* ion content are expected in Ga${}_{1-x}$Y${}_{x}$N and Ga${}_{1-x}$Lu${}_{x}$N systems. The findings presented in this work may encourage further experimental investigations of electronic structures of mixed group III and *RE* nitride materials because, except for Sc-doped GaN and AlN systems, these novel semiconductors were not obtained up to now.

## 1. Introduction

Semiconductor devices based on group III nitrides operate in an exceptionally wide range of energy, e.g., light emitting diodes from the ultraviolet through visible light, up to the infrared region [1,2,3,4]. Solid solutions of group III nitride materials exhibit strong band gap ($E_{g}$) bowings [5,6].

Although rare earth (*RE*) nitrides adopt a rock-salt structure, their relatively narrow band gaps in a range from 0.9 to 1.3 eV [7,8,9,10,11,12] allow one to assume that the introduction of some limited contents of *RE* ions into group III host systems is a promising realization of band gap engineering and can assist in the search for novel nitride semiconductors. A linear decrease in $E_{g}$ with an increasing Sc content was experimentally reveled in Ga${}_{1-x}$Sc${}_{x}$N [13,14,15], Al${}_{1-x}$Sc${}_{x}$N [16,17,18], and Al${}_{1-x}$Y${}_{x}$N [19] alloys. Theoretical investigations followed the experimental research and were focused on Sc-doped GaN [20,21,22] and AlN systems [22].

Calculations based on the density functional theory (DFT) indicated a general tendency in ternary solid solutions of group III and *RE* nitrides to form rock-salt systems [23]. The wurtzite-type materials are expected to be stable for relatively small (less than 0.5) contents of *RE* ions, above which the metastable hexagonal structures of two-dimensional atomic layers of the BN-type are energetically favorable. These predictions are consistent with the findings of previous experimental studies, which were focused on Sc-doped GaN and AlN materials [13,14,15,16,17,18].

Recent investigations of electronic structures of ternary alloys of rock-salt *RE* nitrides revealed very strong band gap bowings in such materials, which are related to *RE* ionic radii mismatch in particular systems [24]. The rock-salt alloys of *RE* and group III nitrides exhibit a linear increase in $E_{g}$ [25], which is also closely connected to the ionic radii of dopant ions despite an opposite relation of band gaps in wurtzite AlN, GaN, and In materials. This may be explained by the fact that valence and conduction band regions of the rock-salt alloys are dominated by the contributions coming from *RE* ions, whereas the contributions of group III ions are located well below and well above the valence band maximum (VBM) and conduction band minimum (CBM) of a material, respectively.

In this work, the structural and electronic properties of wurtzite alloys of group III and *RE* nitrides are predicted from the first principles (DFT-based calculations). The lattice parameters of the materials are studied with the general gradient approximation [26], whereas the fully relativistic band structures are obtained with the use of the Tran–Blacha exchange correlation functional (MBJGGA [27]), which was designed for the accurate studies of semiconductor materials. The discussion of the dependences of $E_{g}$ on *RE* ion contents in the alloys are of particular interest, because, except for Sc-doped GaN and AlN systems, these novel nitride semiconductors were not studied experimentally nor theoretically. The findings presented in this work may encourage further experimental investigations of electronic structures of mixed group III and *RE* nitride materials and their potential applications.

## 2. Results and Discussion

Lattice parameters of parent AlN, GaN, and InN materials, calculated in this work, are gathered in Table 1. As one may expect, the GGA approach yielded slightly overestimated volumes of the unit cells, which is a characteristic feature of this exchange–correlation functional. Similar results were published in previous studies of structural parameters of group III nitrides [20,21,22,28].

**Figure 1:**
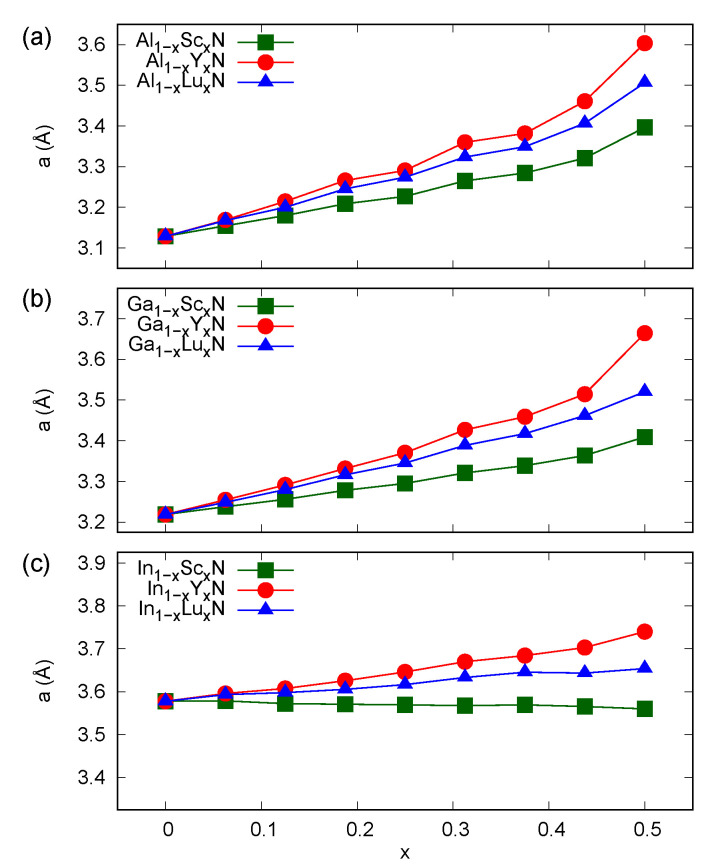
Lattice parameters *a* calculated (GGA) for wurtzite alloys (**a**) Al${}_{1-x}RE_{x}$N, (**b**) Ga${}_{1-x}RE_{x}$N, (**c**) In${}_{1-x}RE_{x}$N, where *RE* = Sc, Y, and Lu.

As presented in Figure 1, the dependencies of a hexagonal lattice parameter *a* on *RE* content in the materials reflect generally bigger ionic radii of RE ions [31], which was also discussed in the previous LDA-based studies for similar rock-salt systems [25]. One may notice an almost negligible lattice mismatch in In${}_{1-x}$Sc${}_{x}$N systems. The lattice parameters in the solid solutions considered here obey the linear Vegard’s law for *x* up to about 0.4, whereas the higher *RE* contents result in a rapid increase in *a*. This effect is particularly evident in the case of the smallest group III ion, i.e., in Al${}_{1-x}RE_{x}$N materials. It is also pronounced in Ga${}_{1-x}$Y${}_{x}$N and In${}_{1-x}$Y${}_{x}$N because of the relatively big ionic radius of yttrium.

**Figure 2:**
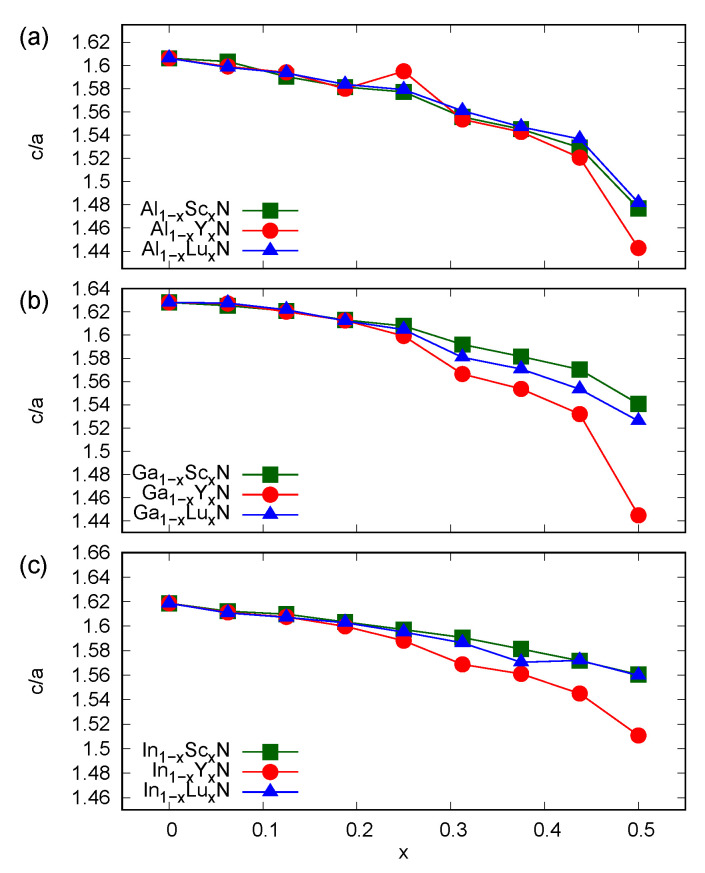
The values of *c/a* ratio calculated (GGA) for wurtzite alloys (**a**) Al${}_{1-x}RE_{x}$N, (**b**) Ga${}_{1-x}RE_{x}$N, (**c**) In${}_{1-x}RE_{x}$N, where *RE* = Sc, Y, and Lu.

The introduction of *RE* ions in group III nitrides results in a tendency to form flattened hexagonal atomic layers, which was suggested in experimental studies for Ga${}_{1-x}$Sc${}_{x}$N systems [14]. This effect was supported by the findings of the recent DFT-based investigations [23], i.e., the full structural relaxation due to the stress tensor and Hellmann–Feynman forces leads to a complete transition between the wurtzite and hexagonal BN type structures in materials with *RE* contents larger than 0.5. The tendency to change the coordination number of ions in mixed nitrides is connected with the various electronic configurations of *d*- (*RE*) and *p*-block (group III) elements. The GGA-derived rapid increase in *a*, presented in Figure 1, is a signature of systems close to the complete flattening of hexagonal atomic layers, which is clearly seen in the *c/a* ratio plots in Figure 2. One may further consider that the alloys with *x* values of less than 0.25 preserve *c/a* values that are very close to those characteristic of pure AlN, GaN, and InN compounds, whereas *x* greater than 0.25 results in more significant modifications to the wurtzite-type structures. Nevertheless, except for the Ga${}_{1-x}RE_{x}$N systems, the rock-salt ground state is expected to be energetically favorable in solid solutions of *RE* and group III nitrides for *RE* contents significantly smaller than 0.5 [23]. Because the available experimental data for high-quality samples were only reported for Sc-doped GaN and AlN [15,18], the issue of structural parameters of hexagonal alloys of *RE*N and group III nitrides requires further experimental investigations.

The band gaps of parent AlN, GaN, and LuN materials, calculated here within the MBJGGA approach, are gathered in Table 1. The $E_{g}$ = 5.12 eV, obtained here for AlN, is lower than the previous MBJLDA results of full and pseudopotential calculations [27,32], which are also lower than the experimental data (6.12 [1]). Similar underestimation of $E_{g}$ is revealed for GaN. A recent study of the electronic structures of group III nitrides reported that the band gaps from the MBJLDA calculations are noticeably smaller than the MBJGGA ones [28]. One may consider some empirical adjustments in the parametrization of the MBJ potential to improve the MBJGGA results for nitride materials [33]. However, such a task is difficult due to the relatively big set of parent compounds studied in this work. The value of $E_{g}$ = 0.7 for InN is in excellent accordance with the experimental data [3,4]. The use of the original MBJ approach is generally desirable in consistent discussion of the results obtained here and reported in the literature.

**Figure 3:**
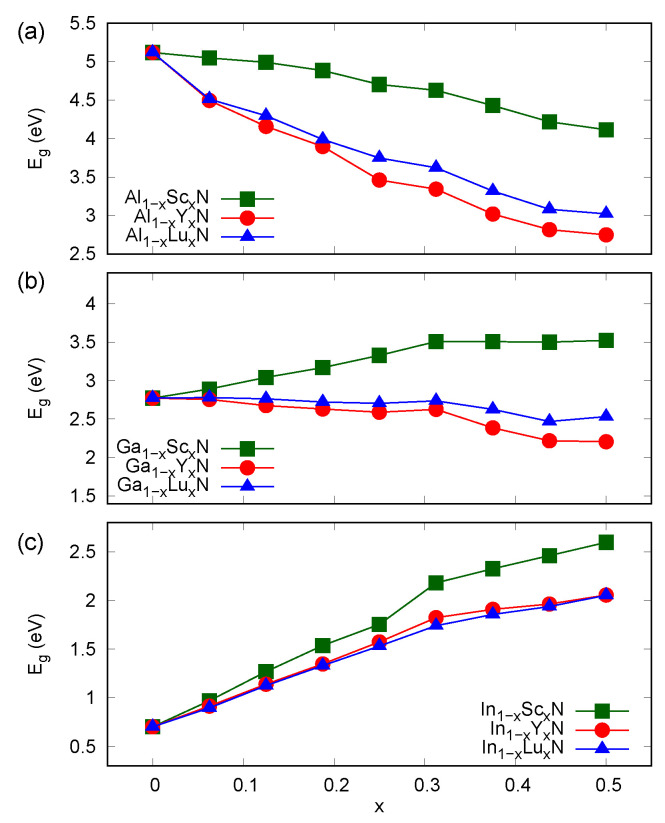
Band gaps calculated (MBJGGA) for wurtzite alloys (**a**) Al${}_{1-x}RE_{x}$N, (**b**) Ga${}_{1-x}RE_{x}$N, (**c**) In${}_{1-x}RE_{x}$N, where *RE* = Sc, Y, and Lu.

The most interesting feature of semiconductor alloys is the band gap engineering. As depicted in Figure 3a, the Al${}_{1-x}$Y${}_{x}$N and Al${}_{1-x}$Lu${}_{x}$N materials may exhibit $E_{g}$ in a wide range with a noticeable bowing. The results presented here are consistent with the available experimental data for Sc- and Y-doped AlN [18,19], taking into account the abovementioned general underestimation of MBJGGA-derived $E_{g}$. A comparable range of $E_{g}$ is available in Al${}_{1-x}$Ga${}_{x}$N alloys [5], whereas smaller band gaps were reported for Al${}_{1-x}$In${}_{x}$N alloys [5]. Therefore, *RE*-doped AlN semiconductors may be expected to be promising materials for applications in the ultraviolet range.

The dependences of $E_{g}$ on *x* in Ga${}_{1-x}RE_{x}$N alloys, as depicted in Figure 3b, are expected to be linear for *x* up to about 0.4, for which some effects connected with structural distortions in hexagonal atomic layers of the materials are revealed. The relatively small change in $E_{g}$ is expected in Y- and Lu-doped GaN systems. The only Ga-based materials that were experimentally studied are Ga${}_{1-x}$Sc${}_{x}$N alloys [13,14,15]. Although the increase in $E_{g}$ with an increasing Sc content in Ga${}_{1-x}$Sc${}_{x}$N is surprising in view of the previous experimental reports [13,14], it has already been demonstrated for high-quality thin films of this material deposited on GaN and AlN buffer layers [15]. This was also explained in previous DFT-based studies [22], which employed the MBJGGA and hybrid exchange–correlation calculations; namely, the hypothetical wurtzite ScN may exhibit $E_{g}$ bigger than 4 eV, which is reflected in an increase of $E_{g}$ in Ga${}_{1-x}$Sc${}_{x}$N systems.

As presented in Figure 3c, the introduction of *RE* ions leads to a strong linear increase of $E_{g}$ in In${}_{1-x}RE_{x}$N systems when compared to that of the InN host material. Comparable band gaps are available in the well-known In${}_{1-x}$Ga${}_{x}$N semiconductors [5]. Similarly to the abovementioned case of Ga${}_{1-x}RE_{x}$N, values of *x* bigger than about 0.3 may induce some structural changes, which affects band gaps in In${}_{1-x}RE_{x}$N alloys. However, this effect is less pronounced in In-based systems due to the fact that the band structures of these semiconductors are dominated by the relatively narrow $E_{g}$ of InN. It is worth recalling that an opposite phenomenon was predicted for the rock-salt *RE*N materials doped with In, i.e, the band gap of such systems increases with increasing In content, as a result of the relatively wide band gap of rock-salt InN [25].

It is worth recalling that the influence of various atomic configurations of alloys on a band gap of group III nitride materials is very strong [5]. The investigations of such effects are beyond the scope of this study. The results presented here were obtained with possibly homogeneous models of alloys because the clustering of *RE* ions is expected to cause a phase segregation in mixed *RE* and group III materials [17].

**Figure 4:**
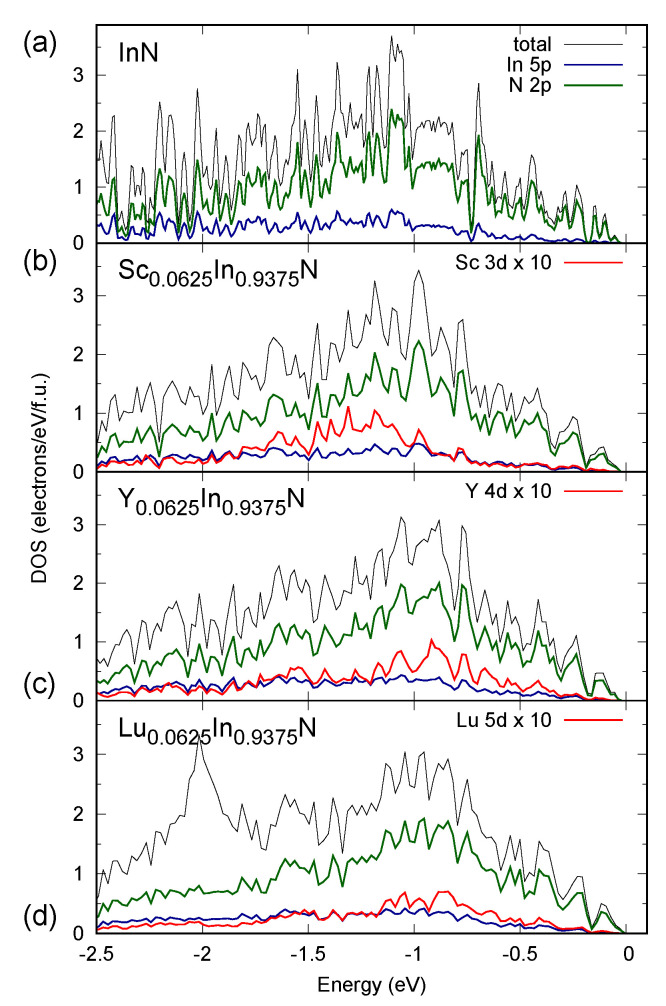
The total and partial density of states (DOS) contributions calculated (MBJGGA) for wurtzite (**a**) InN, (**b**) Sc${}_{0.0625}$In${}_{0.9375}$N, (**c**) Y${}_{0.0625}$In${}_{0.9375}$N, and (**d**) Lu${}_{0.0625}$In${}_{0.9375}$N. Please note that the *d*-type contributions of *RE* ions are magnified by 10.

A careful analysis of total and partial contributions into the density of states (DOS) in the vicinity of VBM in *RE*-doped InN materials, presented in Figure 4, reveals some common features of semiconducting nitrides. Namely, the valence regions of these materials are mainly formed by the N 2p states and some minor contributions of the *p* and *d* states coming from group and *RE* ions, respectively. The characteristic electronic structure of nitrides near VBM is unaffected by the doping, which was also found for the materials in an opposite regime of compositions, i.e., the rock-salt alloys of Al/Ga/In-doped *RE*N systems [25]. The unoccupied *d*-electron contributions coming from *RE* ions are located above the CBM region (not shown). The evolution of a band gap in the WZ alloys is connected with some chemical pressure related to the relatively big ionic radii of *RE* elements and the presence of the *d*-type contributions into the total DOS in the vicinity of CBM of a host material.

## 3. Conclusions

The structural properties of solid solutions of *RE* and group III nitrides predicted from first principles are rather complex. Similarly to the findings of some experimental studies for Sc-doped GaN, one may observe a flattening of the wurtzite atomic layers, which is directly connected with the presence of the *RE* ion in the material. The GGA-based results indicate rather small structural modifications for contents of *RE* lower than 0.25, whereas a very rapid change in the *c/a* ratio was found for *RE* contents close to one half. Because this effect is the most pronounced in Y-doped systems, the size of the *RE* ion may be regarded as an important factor for the abovementioned structural modification.

The decreasing band gap as a function of *x* is expected in Al${}_{1-x}RE_{x}$N materials. The strong bowing of $E_{g}$ was found for Al${}_{1-x}$Y${}_{x}$N and Al${}_{1-x}$Lu${}_{x}$N. Smaller reductions of $E_{g}$ were obtained for Ga-based materials, except for Ga${}_{1-x}$Sc${}_{x}$N, in which $E_{g}$ increased with an increasing Sc content. The doping with *RE* ions also seems to be a reasonable strategy of band gap widening in InN. The electronic structure of this family of materials is especially interesting due to the complete lack of any experimental reports on *RE* doped InN systems. The results presented in this work may encourage further experimental investigations of structural and electronic properties of novel nitride semiconductor alloys.

## 4. Materials and Methods

The DFT calculations were performed using the VASP package [34,35]. The plane wave augmented (PAW [36]) atomic datasets with Perdew–Burke–Ernzerhof parameterization (GGA [26]) of the exchange–correlation functional were employed. The solid solutions were modeled with 2 × 2 × 2 supercells, i.e., the multiplications of the wurtzite primitive cell. Possibly homogeneous atomic configurations were selected. All structural properties, i.e., lattice parameters and atomic positions, were fully relaxed via stresses/forces optimization. The 500 eV plane-wave energy cutoff and 6 × 6 × 6 **k**-point lattice were selected. The band structures and DOS plots were obtained in the fully relativistic mode within the MBJGGA approach [27]. 

## Figures and Tables

**Table 1:** Hexagonal lattice parameters *a* and *c/a* ratios (GGA), and band gaps (MBJGGA) calculated in this work, and available experimental data for wurtzite group III nitrides.

Compound	*a* (Å)	c/a	$E_{g}$ (eV)
AlN this work	3.129	1.606	5.12
AlN experim.	3.110 [29]	1.601 [29]	6.12 [1]
GaN this work	3.219	1.629	2.77
GaN experim.	3.190 [29]	1.627 [29]	3.50 [2]
InN this work	3.578	1.619	0.70
InN experim.	3.544 [30]	1.613 [30]	0.69 [4]

## Data Availability

The data presented in this study are available on reasonable request from the corresponding author.
